# Network pharmacology and molecular docking predictions of the active compounds and mechanism of action of Huangkui capsule for the treatment of idiopathic membranous nephropathy

**DOI:** 10.1097/MD.0000000000035214

**Published:** 2023-09-15

**Authors:** Meng Cai, Yongjing Xiang, Zhengsheng Li, Juan Xie, Fulong Wen

**Affiliations:** a Nephrology Department, Secondary Affiliated Hospital of Guizhou University of Traditional Chinese Medicine, Guiyang, China.

**Keywords:** gene ontology, Huangkui, idiopathic membranous nephropathy, network pharmacology

## Abstract

**Background:**

Huangkui Capsule is a single herbal concoction prepared from the flower of Abelmoschus manihot, which is used to treat idiopathic membranous nephropathy (IMN), a frequent pathologically damaging kidney condition. It has been widely utilized to treat a variety of renal disorders, including IMN, in clinical practice. However, the active compounds and mechanism of action underlying the anti-IMN effects of Huangkui Capsule remain unclear. In this study, we aimed to predict the potential active compounds and molecular targets of Huangkui Capsule for the treatment of IMN.

**Methods:**

The possible active components of Huangkui were located using the SymMap v2 database. The targets of these drugs were predicted using Swiss Target Prediction, while IMN-related genes with association scores under 5 were gathered from the GeneCards and DisGeNET databases. The common targets of the disease and the components were determined using VENNY 2.1. Using Cytoscape 3.8.0, a drug-disease network diagram was created. Molecular docking was carried out with Pymol, AutoDock Tools, and AutoDock Vina.

**Results:**

With 1260 IMN-related illness genes gathered from GeneCards and DisGeNET databases, we were able to identify 5 potentially active chemicals and their 169 target proteins in Huangkui. Based on degree value, the top 6 targets for Huangkui treatment of IMN were chosen, including AKT, MAPK3, PPARG, MMP9, ESR1, and KDR.

**Conclusion:**

This work theoretically explains the mechanism of action of Huangkui Capsule in treating IMN and offers a foundation for using Huangkui Capsule in treating IMN in clinical settings. The findings require additional experimental validation.

## 1. Introduction

One of the most common glomerular disorders in adults is Membranous Nephropathy, a condition characterized by glomerular damage brought on by autoimmune responses. It is known as primary membranous nephropathy or idiopathic membranous nephropathy (IMN) and affects about 80% of MN cases.^[1]^ Its traits include the thickening of the kidney’s capillary wall, modifications to the basement membrane, and the presence of substantial immune deposits in the subepithelial area.^[2]^ The incidence of IMN, the most prevalent pathogenic form of adult nephrotic syndrome, is rising in China and is one of the major causes of renal failure.^[3]^ Approximately 1 to 3rd of IMN patients experience a spontaneous remission, 1 to 3rd experience chronic proteinuria and long-term preservation of renal function, and the remaining 1 to 3rd experience a steady progression of the disease till end-stage renal disease. As a result, the prognosis of the condition depends on how IMN patients are treated.^[4]^ Immunosuppressive therapy, which involves the use of numerous hormone drugs to weaken the patient’s own immunity in order to diminish proteinuria and postpone the future development of IMN, is currently the most common form of treatment. However, it is impossible to overlook the negative effects of hormone therapy, and IMN following hormone therapy is still prone to relapse.^[5]^

Over the course of thousands of years, Traditional Chinese Medicine (TCM) has amassed invaluable information in the fight against diseases. Water hibiscus, also known as huangkui (Abelmoschus manihot), has a history that dates back to Ge Hong “Prescriptions for Emergent Reference” in the Eastern Jin Dynasty (3–4th century AD), or roughly 2000 years ago. In China, the ethanol extract of Huangkui is used to make the TCM supplement known as the Huangkui capsule, which is used to treat renal illnesses such as IMN. In China, the ethanol extract of Huangkui is used to make the TCM supplement known as the Huangkui capsule, which is used to treat renal illnesses such as IMN.^[6]^ Huangkui capsule may treat kidney diseases by reducing inflammation and oxidative stress, enhancing immune responses, protecting renal tubular epithelial cells, reducing podocyte apoptosis, glomerulosclerosis, and mesangial proliferation, and inhibiting renal fibrosis, according to recent pharmacological studies.^[7,8]^ However, little is known about the chemical constituents and mechanisms underlying Huangkui anti-IMN actions. With its Holistic Concept, TCM diagnosis and treatment philosophy has spawned a new wave of research models that are defined by “network” and “system” aspects. To comprehend the multi-target and multi-pathway processes of TCM, emerging network pharmacology and molecular docking approaches are now frequently employed to determine the effective components of medications.^[9,10]^ Therefore, in this study, we used network pharmacology and molecular docking techniques to predict the potential active ingredients, molecular targets, and signaling pathways of Huangkui against IMN.

## 2. Materials and methods

### 2.1. Collection and screening of potential active compounds from Huangkui

A database of TCM syndrome correlations called Symptom Mapping (SymMap v2, http://www.symmap.org/) contains a large number of herbs, components, and matching TCM symptoms that are closely related to modern medicine (MM) symptom words. Through symptom-disease or constituent-target interactions, SymMap also gathers the targets (genes) and diseases linked to these herbs, totaling 4302 targets and 5235 disorders. As a result, SymMap offers a wealth of descriptive data on herbs, TCM symptoms, MM symptoms, ingredients, targets, and disorders. Additionally, it gives pairwise correlations between all 6 types of components, including associations between herbs, TCM syndrome, MM symptoms, constituent diseases, and targets, using either direct or indirect statistical inference. This links TCM and modern medicine from the phenotypic to the molecular levels.^[11]^ The keyword “Abelmoschus manihot” was used to do a search in SymMap, and its component parts were exported.

The Traditional Chinese Medicine Systems Pharmacology database and analysis platform (TCMSP v2.3, https://old.tcmsp-e.com/load_intro.php?id=33) includes information on the absorption, distribution, metabolism, and excretion characteristics of each compound. Oral bioavailability (OB) is a term that refers to “the rate and extent of absorption of the active ingredient or active part from the therapeutic product, which is available at the target site “. Drug-likeness (DL) is a qualitative paradigm for drug design that combines the ADME (Absorption, Distribution, Metabolism, and Excretion) qualities of ingredients and existing drugs.^[12]^ In the TCMSP database, potential active compounds from Huangkui were collected by searching for compounds that simultaneously meet the requirements of OB ≥ 30% and DL ≥ 0.18 for subsequent target prediction.

### 2.2. Target prediction of potential active compounds in the herb Huangkui

A search for possible Huangkui active ingredients was carried out in PubChem (https://pubchem.ncbi.nlm.nih.gov), a freely accessible open database initiative funded by the National Institutes of Health and built on the bioinformatics platform of the National Center for Biotechnology Information. A significant amount of experimental structural and biochemical data on small molecule drugs is kept in PubChem.^[13]^ The SMILES formula was discovered while scanning Pubchem for maybe active Huangkui compounds.

Swiss Target Prediction can predict the targets of compounds based on similarities to the 2-dimensional and 3-dimensional structure of known compounds. The known compound-target interactions come from the ChEMBL database, which contains interactions between 280,381 small molecules and 2686 targets, with most targets (66%) being human proteins. Swiss Target Prediction provides a score for each predicted target to assess the likelihood of correct prediction. It also makes predictions based on homology mapping between different species and provides probability scores for correctness.^[14]^ Swiss Target Prediction was used to predict the targets of action of the potentially active compounds of Huangkui using the SMILES formulas of the potentially active compounds retrieved from Pubchem.

### 2.3. Collection and screening of genes associated with IMN

DisGeNet (https://www.disgenet.org/) is a database that specializes in gathering data on human genes and mutation sites associated with diseases.^[15]^ Gene Cards, a comprehensive database of human genes, may be found at https://www.genecards.org. It offers short details on all identified and expected human genes in relation to genomics, proteomics, transcription, genetics, and function. Gene Cards provide useful data, such as associations with illnesses, polymorphisms and mutations, gene expression, gene function, pathways, protein-protein interactions, and more.^[16]^

We searched and gathered genes in the DisGeNet and Gene Cards databases using the phrase “Idiopathic Membranous Nephropathy” and chose genes with a relevance score of 5 as disease-associated targets for IMN.

### 2.4. Screening of potential targets of Huangkui for treating IMN

IMN-related targets were compared to the putative targets of Huangkui active components, and the intersection of the 2 sets was discovered using the Venny 2.1.0 online tool (https://bioinfogp.cnb.csic.es/tools/venny/index.html). Huangkui prospective targets for treating IMN are represented by the resulting collection of common targets.

### 2.5. Protein-protein interaction analysis

The relationship between proteins and their functions, as is widely known, is the basis of cellular systems. Proteins are the primary carriers of all biological activity. We used the Search Tool for the Retrieval of Interaction Gene/Proteins (String, https://cn.string-db.org/) to analyze the potential targets of Huangkui for treating IMN, limited to Homo sapiens, with a minimum interaction score set at medium confidence (0.400), and hidden nodes without connections in the network. This analysis allowed us to fully understand biological phenomena and identify the relationships among the 67 potential targets of Huangkui for treating IMN. The protein-protein interactions (PPI) were gathered. The String database, a database for searching protein-protein interactions, was created by Peer Bork team at the European Molecular Biology Laboratory on the functional link between genes. It encompasses both indirect functional correlations between proteins and their direct physical interactions. Along with experimental data, outcomes gleaned from PubMed abstracts, and combined information from other sources, it also contains outcomes foreseen using bioinformatics techniques.^[17,18]^ Cytoscape software (version 3.8.0) was used to further visualize the results of the PPI study.

### 2.6. Gene ontology (GO) functional enrichment analysis and Kyoto encyclopedia of genes and genomes (KEGG) pathway enrichment analysis

GO and KEGG enrichment analysis are common tools for analyzing genes.^[19]^ GO is a database established by the Gene Ontology Consortium, with the aim of creating a semantic vocabulary standard applicable to various species to describe and limit the functions of genes and proteins, which can be updated with further research. GO defines a set of dynamic controlled vocabularies to describe the roles of genes and proteins in cells, thereby comprehensively describing the attributes of genes and gene products in organisms. GO functional enrichment analysis includes 3 components: biological process (BP), cellular component (CC), and molecular function,^[20]^ which can help identify basic genes in specific organisms to facilitate the study of their basic roles in living organisms. KEGG is a manually curated database resource that integrates various biological objects classified into systems, genomes, chemistry, and health information.^[21]^ The core pathways revealed by KEGG enrichment analysis and the connections between these basic genes can further help us understand the key roles of these genes. GO and KEGG enrichment analysis were performed using the DAVID Bioinformatics Resources database, which was developed by the Laboratory of Human Retrovirology and Immunoinformatics.^[22]^ Running the database yielded the results of the GO functional enrichment analysis and KEGG pathway enrichment analysis after importing the target genes of Huangkui for IMN and choosing the species as humans.

### 2.7. Molecular docking

Molecular docking is a widely used in drug discovery that aims to predict the binding conformation of small molecule ligands to appropriate target binding sites. Docking can identify new compounds with therapeutic potential, predict ligand-target interactions at the molecular level, or depict structure-activity relationships without prior knowledge of the chemical structures of other target modulators.^[23,24]^ The PubChem database was searched for probable Huangkui active ingredients, and their 2D structures were obtained in SDF format for this investigation. ChemDraw V20 and Chem3D were used to convert the structures into 3D structures and save them in pdb format. Using Auto Dock Tools 1.5.7, the compounds were transformed into ligands by adding charges before being exported in pdpqt format for future molecular docking. The protein sequence database Uniprot (https://www.uniprot.org) was used to identify the core target, and the Protein Data Bank (https://www.rcsb.org) was used to locate the related 3D crystal structure. In pymol, the 3D structure was opened, and extra chains, ions, and water molecules were taken out. Auto Dock Tools 1.5.7 was then used to convert the protein into pdbqt format for molecular docking. The macromolecule (core target protein) and ligand (potentially active compound) structures were successively imported into AutoDockTools 1.5.7 in the pdbqt format, the Grid Box parameters were configured and saved, AutoDock Vina 1.2.3 was used to perform molecular docking, and the outcomes were exported. Pymol 2.5 was used to visualize and analyze the complex of the core target protein and the ligand following molecular docking in order to determine their relative binding affinities.

## 3. Result

### 3.1. Potential active compounds of Huangkui

In SymMap v2, 29 elements were connected to Huangkui. Five possible active compounds were discovered following screening in TCMSP using the criterion of OB ≥ 30% and DL ≥ 0.18, as shown in Table 1.

**Table 1:** Potential active ingredients of Huangkui capsule.

CAS ID	Ingredient name	Molecular Formula	OB (%)	DL
117-39-5	Quercetin	C15H10O7	46.43	0.28
68555-08-8	Stigmasterol	C29H48O	43.83	0.76
481-18-5	α-spinasterol	C29H48O	42.98	0.76
489-35-0	Gossypetin	C15H10O8	35	0.31
5779-62-4	β-sitosterol	C29H50O	33.94	0.7

DL = drug-likeness, OB = oral bioavailability.

### 3.2. Targets of potential active ingredients in Huangkui capsules

**Figure 1.**
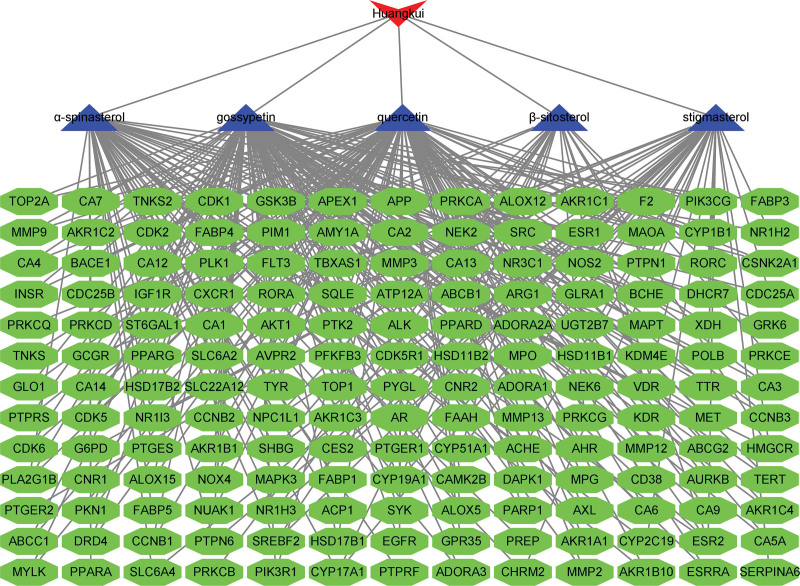
Huangkui capsules-ingredients-targets network diagram disease related genes.

According to the Swiss Target Prediction website, the 5 possible active Ingredients could each affect 500 different targets. Setting a threshold of probability > 0 and eliminating duplicate targets among the 5 Ingredients allowed for further selection, leaving 169 viable targets for additional study, as shown in Figure 1.

### 3.3. Disease related genes

2316 IMN-related genes and 79 IMN-related genes were found in the Gene Cards database and DisGeNET, respectively. We ultimately found 1260 IMN-related genes by restricting the Relevance Score ≥ 5 and taking the intersection.

### 3.4. Potential targets of Huangkui capsule in treating IMN

**Figure 2.**
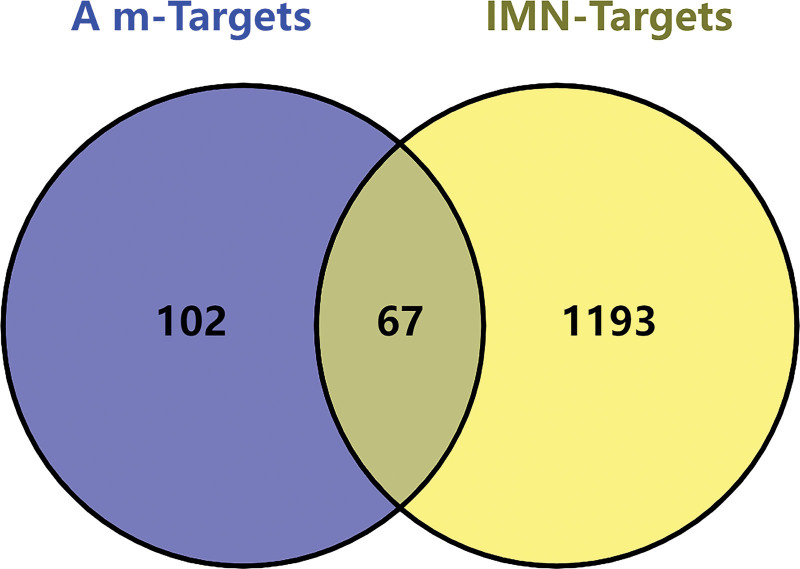
VENNY diagram illustrating the intersection between targets of Huangkui and targets associated with IMN. IMN = idiopathic membranous nephropathy.

The possible targets of Huangkui for the treatment of IMN are depicted in Figure 2 and include 67 common targets between the active components prospective targets and IMN-related targets.

### 3.5. Protein-protein interaction analysis

**Figure 3.**
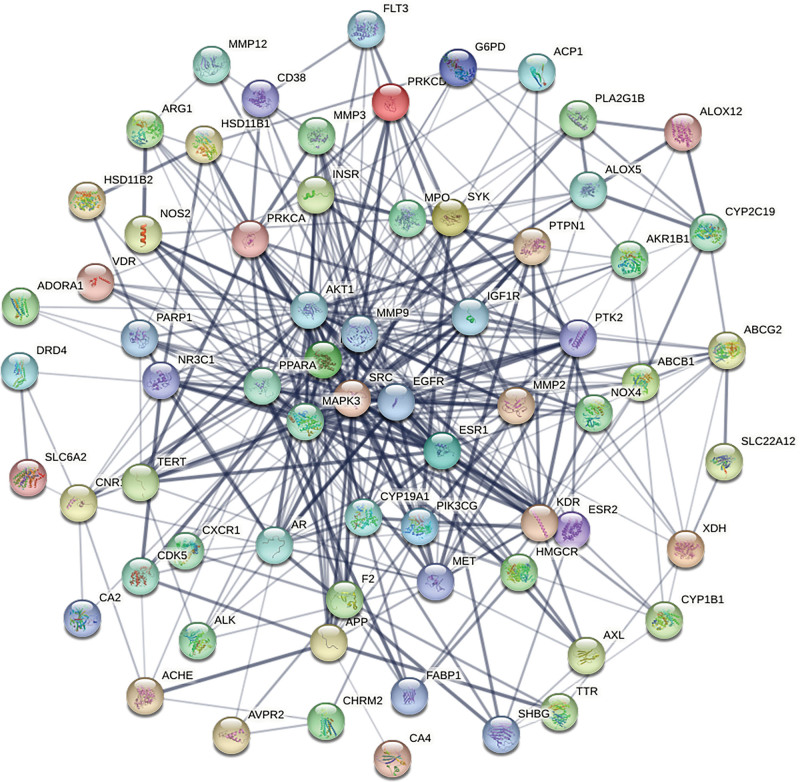
PPI interaction network of 67 potential target proteins of Huangkui capsule for the treatment of IMN constructed using the string database. The thickness of the lines indicates the strength of the data support. IMN = idiopathic membranous nephropathy, PPI = protein-protein interactions.

In order to create the PPI network depicted in Figure 3, the 67 putative targets for treating IMN identified from the herb Huangkui were loaded into the String database. With 67 nodes and 383 edges, the PPI network has an average node degree of 11.4, an average local clustering coefficient of 0.525, and *P* value < 1.0E-16.

**Figure 4.**
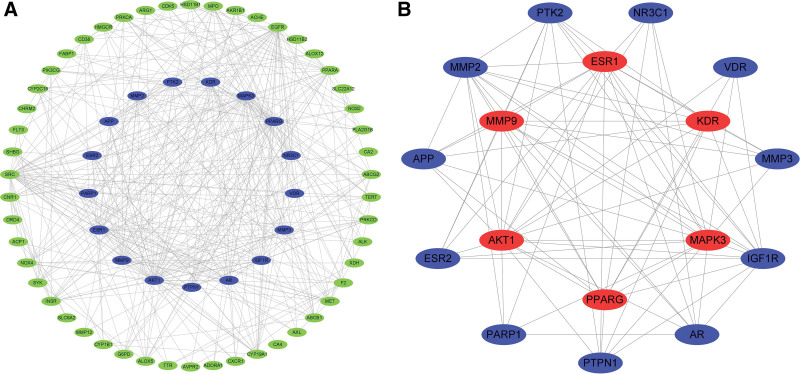
PPI interaction network of 17 core anti-IMN targets selected by Cytoscape 3.8.0 and the 67 potential anti-IMN targets of Huangkui capsule. The nodes in red have the highest degree of interaction. IMN = idiopathic membranous nephropathy, PPI = protein-protein interactions.

The Cytoscape software version 3.8.0 (https://cytoscape.org/) was used to import the TSV-formatted results of the String database analysis. AKT1, MAPK3, PPARG, MMP9, ESR1, and KDR were chosen as the key targets of Huangkui for the therapy of IMN (Fig. 4B) 6 after analysis using the MCODE plugin (Fig. 4A) (Table 2). On those 6 core targets, molecular docking was carried out.

**Table 2:** Core targets of Huangkui capsule for treating IMN.

Uniprot	Gene	Protein	PDB ID	Degree
P31749	AKT1	RAC-alpha serine/threonine-protein kinase	1H10	41
Q16644	MAPK3	MAP kinase-activated protein kinase 3	3FHR	37
P37231	PPARG	Peroxisome proliferator-activated receptor gamma	1FM6	31
P14780	MMP9	Matrix metalloproteinase-9	1GKC	28
P03372	ESR1	Estrogen receptor	1A52	27
P35968	KDR	Vascular endothelial growth factor receptor-2	1VR2	20

IMN = idiopathic membranous nephropathy.

### 3.6. GO functional enrichment analysis

**Figure 5.**
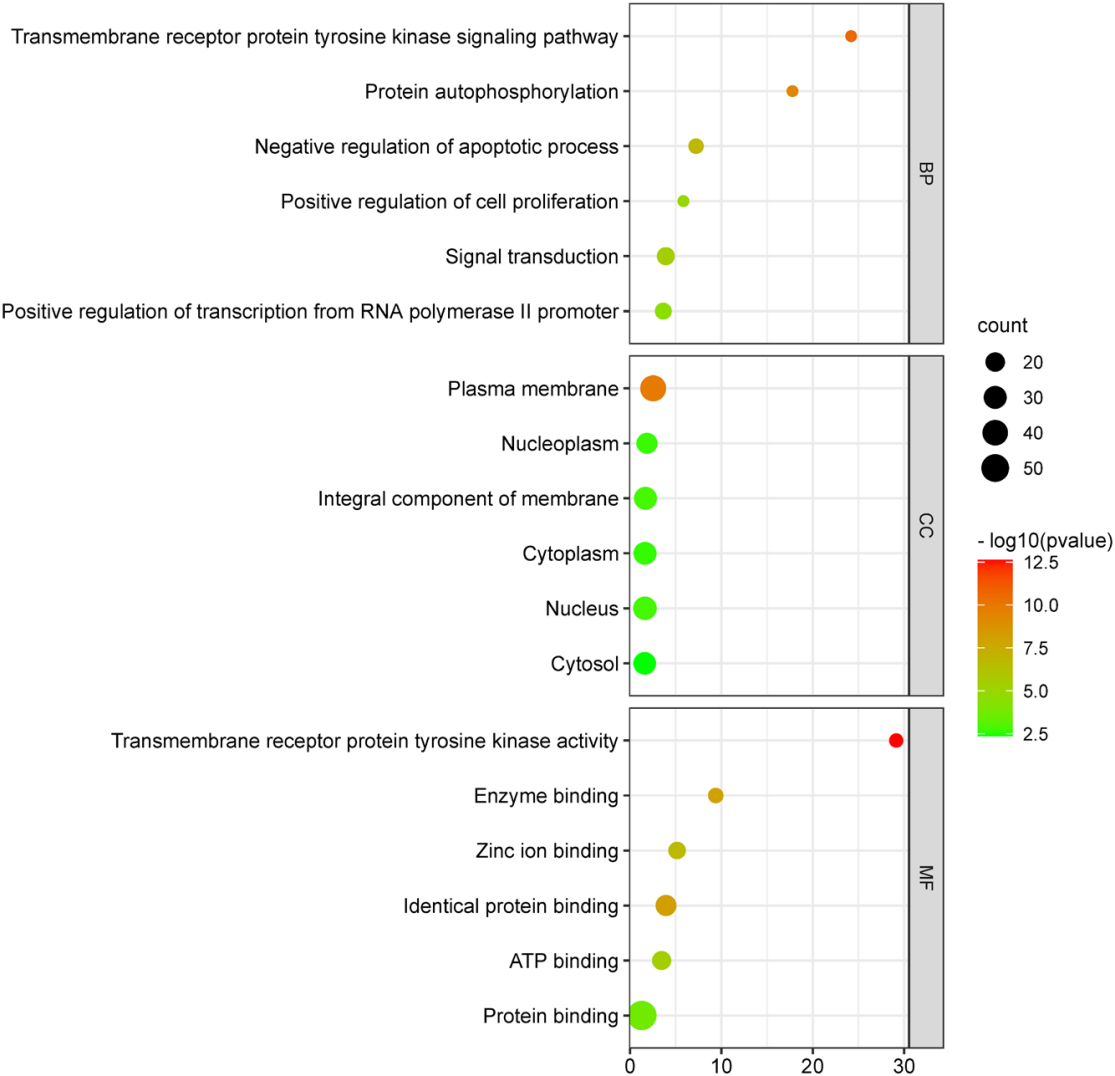
GO enrichment analysis of the 67 anti-IMN targets of Huangkui capsule. The x-axis represents the number of targets, while the y-axis shows the enriched biological processes (BP), cellular components (CC), and molecular functions (MF) (top 6 ranking). GO = gene ontology, IMN = idiopathic membranous nephropathy.

**Figure 6.**
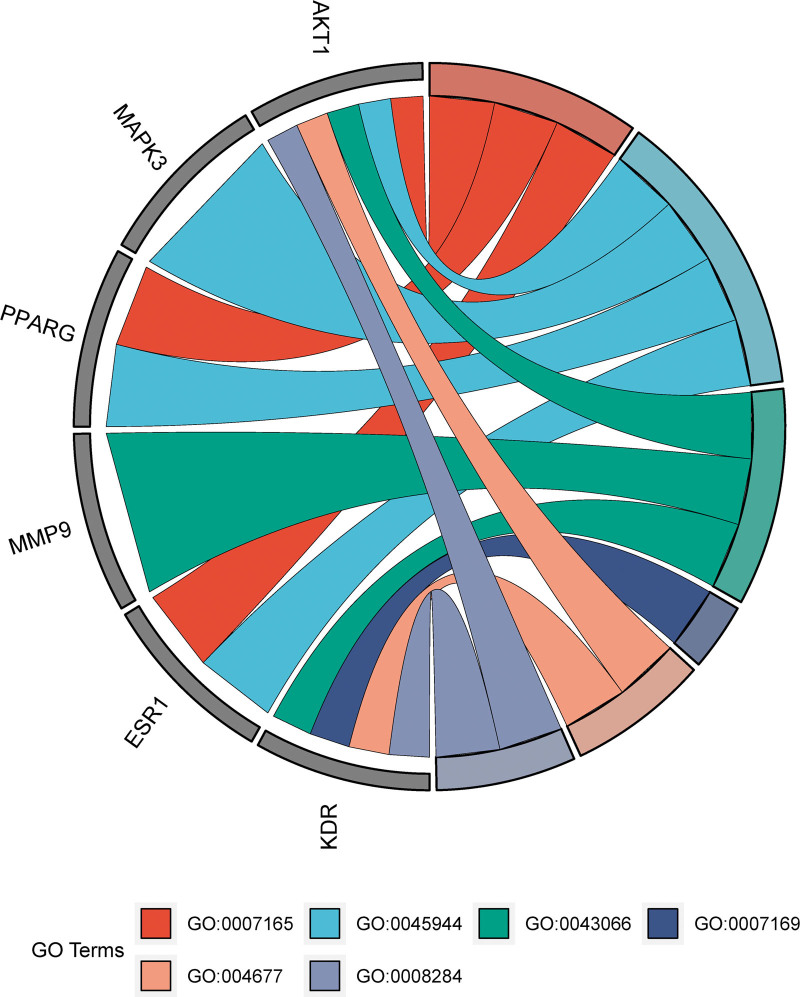
GO enrichment analysis of 6 core anti-IMN targets of Huangkui capsule. GO = gene ontology, IMN = idiopathic membranous nephropathy.

The 67 IMN-related targets of Huangkui that we examined using GO functional enrichment analysis revealed that these genes are engaged in 313 biological processes, 50 CCs, and 87 molecular activities. To visualize the top 6 terms in each category, we chose a bubble chart (Fig. 5). The targets of Huangkui in treating IMN are involved in a variety of BP, including signal transduction, positive regulation of RNA polymerase II transcription, negative regulation of apoptosis, transmembrane receptor protein tyrosine kinase signaling pathway, protein phosphorylation, and positive regulation of cell proliferation, among others. These targets were revealed by the results of GO functional enrichment analysis. Targets related to CC include the cytoplasmic membrane, nucleus, and cytoplasm. Protein binding, homodimerization, ATP binding, zinc ion binding, transmembrane receptor protein tyrosine kinase activity, and other processes are among the enriched molecular functions. We also depicted the connections between the top 6 BP and the 6 main targets (Fig. 6).

### 3.7. KEGG pathway enrichment analysis

**Figure 7.**
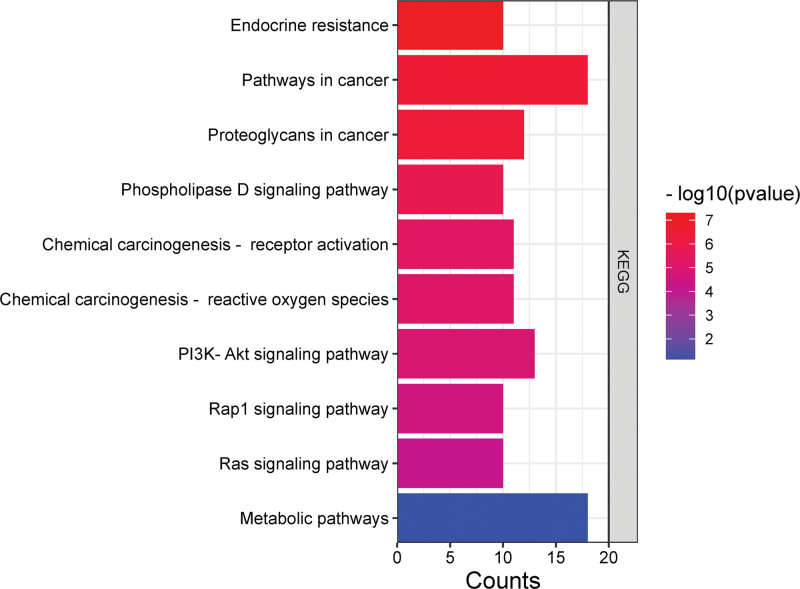
KEGG pathway enrichment analysis of the 67 anti-IMN targets of Huangkui capsule. The x-axis represents the number of genes, and the y-axis represents various KEGG pathways (top 10). IMN = idiopathic membranous nephropathy, KEGG = Kyoto encyclopedia of genes and genomes.

**Figure 8.**
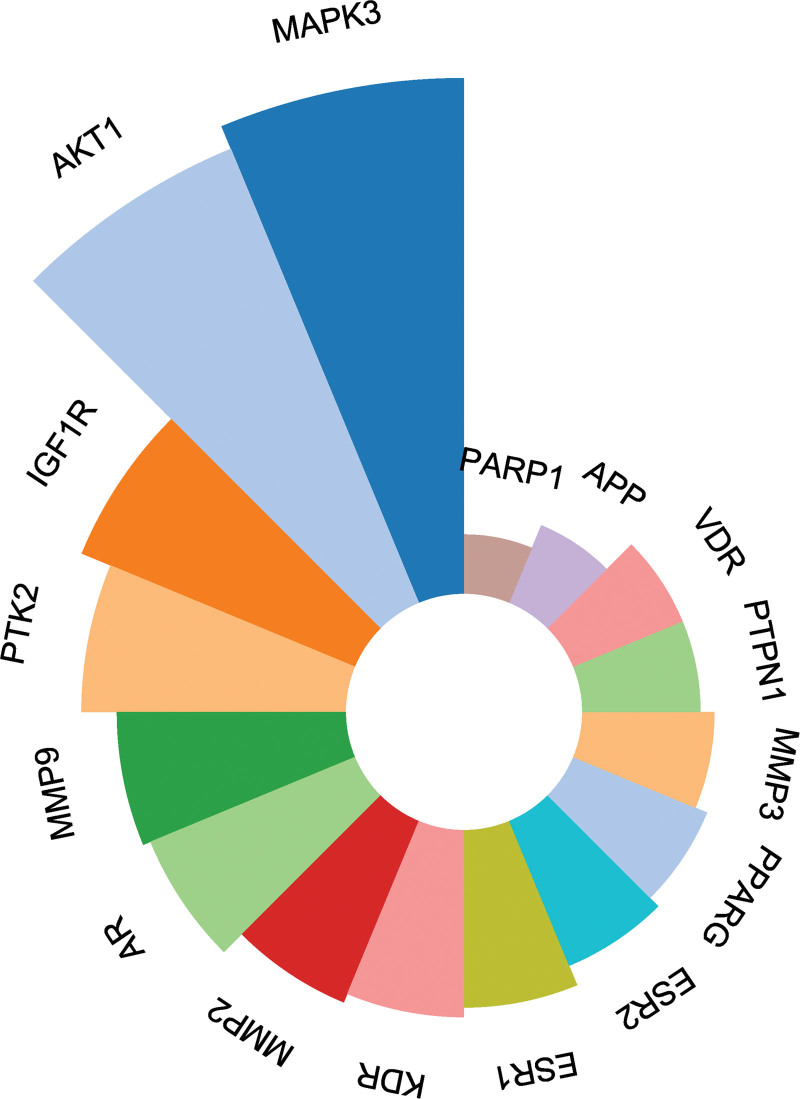
Number of Signaling Pathways Involved in 17 Target Genes. NCR1 did not enrich in any pathway.

**Figure 9.**
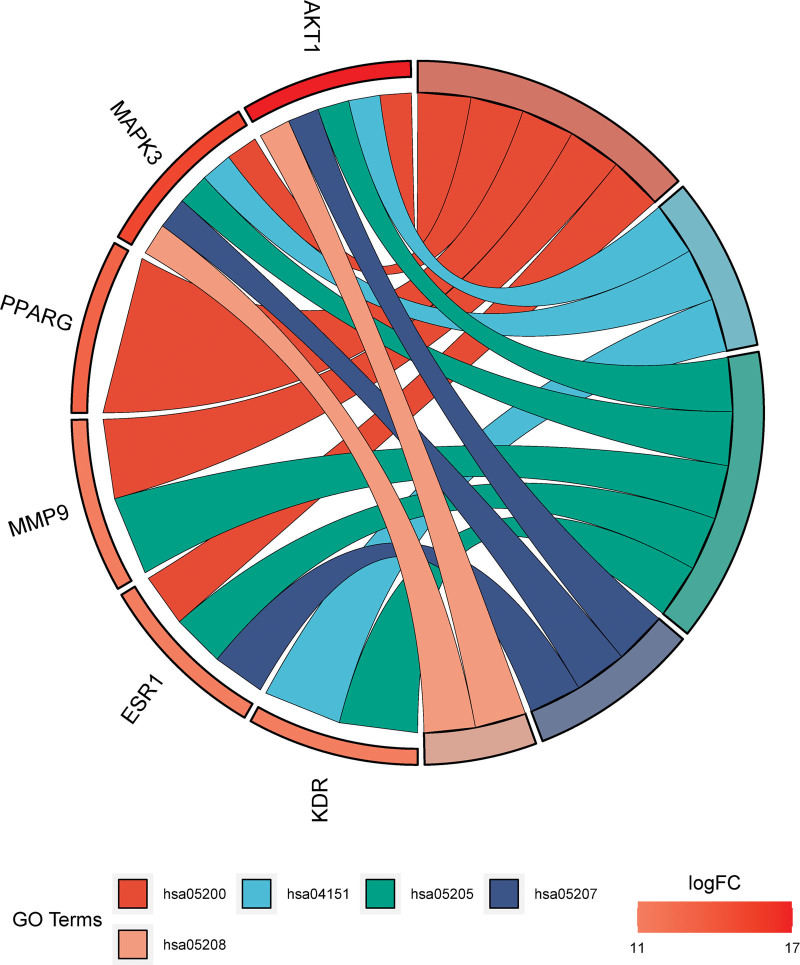
Relationship between 6 core targets and the top 6 enriched signaling pathways in KEGG analysis. KEGG = Kyoto encyclopedia of genes and genomes.

The signaling pathways used in Huangkui treatment of IMN were anticipated based on KEGG pathway enrichment analysis. The DAVID Bioinformatics Resources database revealed that the 67 overlapping targets were implicated in 99 signaling pathways, with *P* < .05 for each pathway. Figure 7 displays the top 10 KEGG pathways ordered by count. The KEGG pathway enrichment analysis results revealed that the main phosphatidylinositol 3-kinase (PI3K)-Akt signaling pathway, metabolic pathways, cancer pathways, proteoglycans in cancer, chemical carcinogenesis-receptor activation pathway, and chemical carcinogenesis-ROS pathway were among Huangkui targets in the treatment of IMN. These anticipated routes might be Huangkui signaling pathways for the therapy of IMN. Create a chord plot (Fig. 8) between the 6 core targets and the top 6 KEGG pathways and a polar plot (Fig. 9) between the 17 selected targets and the associated enriched KEGG pathways (the hsa01100 metabolic pathway will not be displayed because it has not enriched to 6 core targets).

### 3.8. Molecular docking

**Figure 10.**
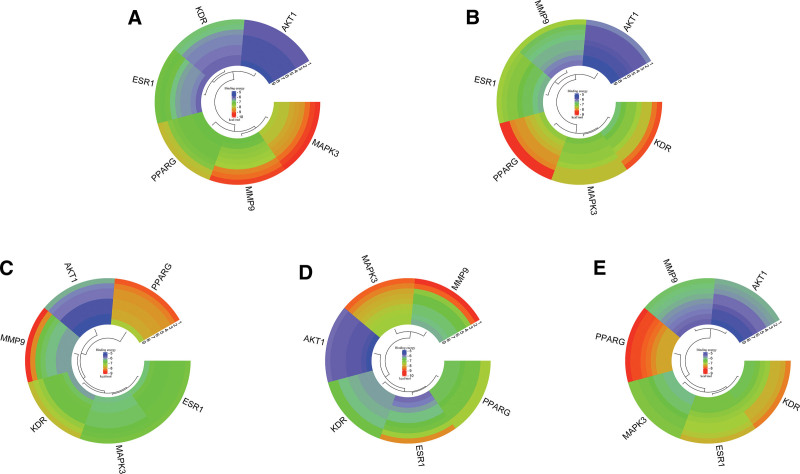
Circular clustering map of molecular docking binding energies between 5 potential active compounds and 6 core targets. (A) The molecular docking results of quercetin with 6 core targets. (B) The molecular docking results of stigmasterol with the 6 core targets. (C) The molecular docking results of α-spinasterol with 6 core targets. (D) The molecular docking results s of gossypetin with the 6 core targets. (E) The molecular docking results of β-sitosterol with 6 core targets.

**Figure 11.**
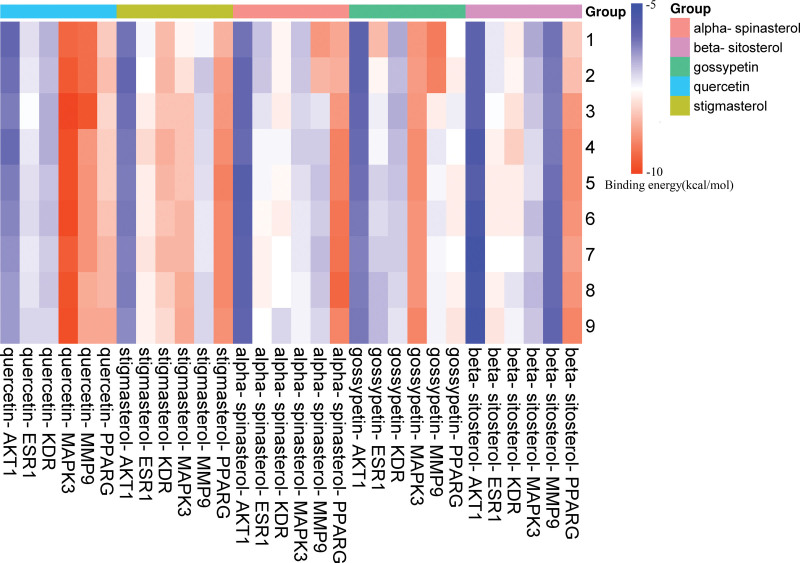
Heat map of molecular docking binding energies.

**Figure 12.**
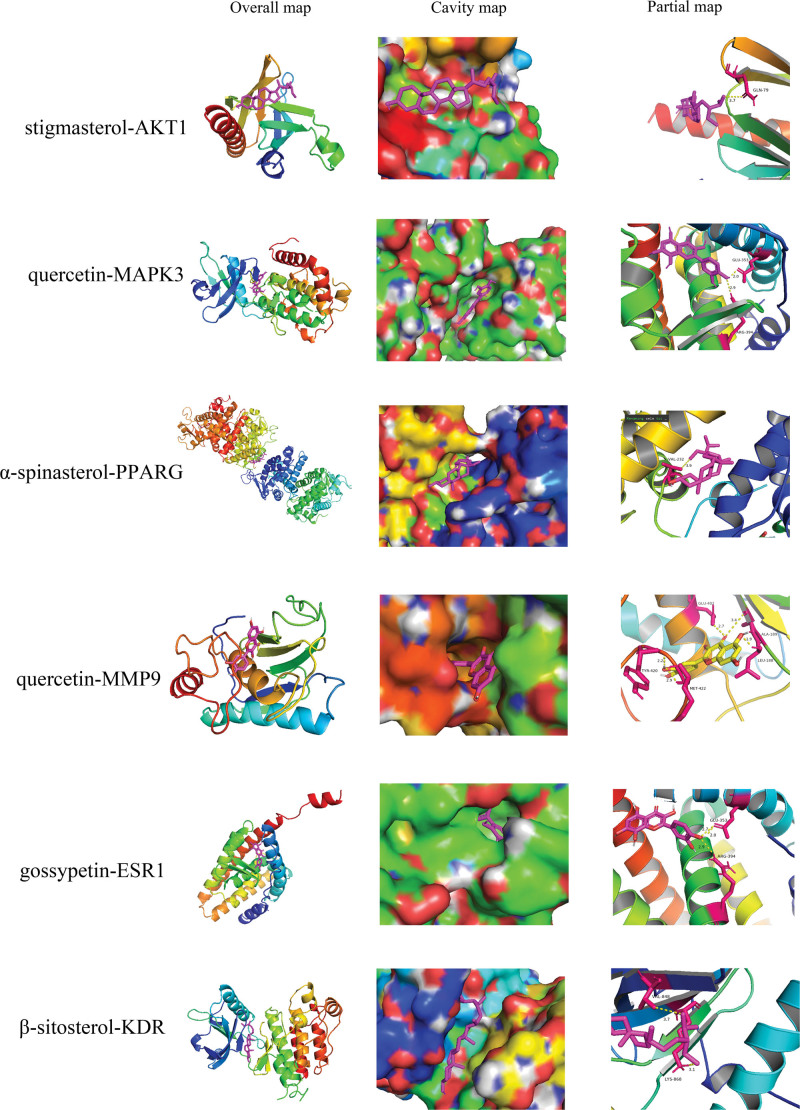
Display of partial components and molecular docking results.

AKT1, MAPK3, PPARG, MMP9, ESR1, and KDR, which are the main targets of Huangkui in the treatment of IMN, were docked with 5 of its putative active components sequentially. Binding energy ratings were used to indicate the binding affinity between the proteins and active ingredients (ligands). A smaller value denotes a tighter and more substantial binding. Typically, a value of < 1.2 kcal/mol is considered relevant for docking. For each core target and component docking, the highest 9 sets of binding energy scores were chosen and displayed in a circular cluster map (Fig. 10). The findings demonstrated that the possible active ingredients in huang kui were strongly bound to the 6 targets of AKT1, MAPK3, PPARG, MMP9, ESR1, and KDR, indicating that these targets may be directly acted upon by the active ingredients in Huang Kui to have therapeutic effects on IMN. The outcomes of the prior network pharmacology investigation were further supported by the molecular docking data. Based on the number of compounds, the compounds were separated into 5 groups, and a heatmap was created to compare the binding energies of the compounds with each target on a horizontal scale (Fig. 11). Using global, cavity, and local maps, the lowest binding energy between each core target and chemical was chosen for display (see Fig. 12), which were, respectively, stigmasterol-AKT1 (−6.3 kcal/mol), quercetin-MAPK3 (−9.4 kcal/mol),α-spinasterol-PPARG (−8.6 kcal/mol), quercetin-MMP9 (−9.3 kcal/mol), gossypetin-ESR1(−8.4 kcal/mol), and β-sitosterol-KDR (−7.8 kcal/mol).

## 4. Discussion

**Figure 13.**
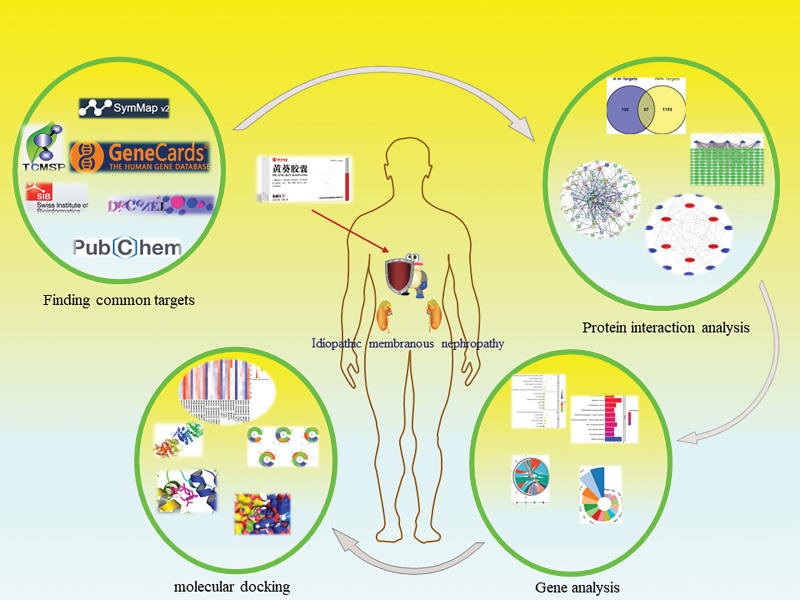
Network pharmacology and molecular docking process of Huangkui capsule in the treatment of idiopathic membranous nephropathy.

The therapeutic effects of the Huangkui capsule, a traditional Prepared Chinese Medicine frequently used in clinical practice to treat renal disease, on diabetic nephropathy have been thoroughly investigated.^[25–28]^ On the other hand, there is not a lot of study on its application in the management of IMN. Therefore, this study analyzes the potential active ingredients and potential molecular mechanisms of Huangkui capsule in the treatment of IMN based on systems biology theory and molecular docking technology (Fig. 13). It is known that circulating autoantibodies attach to 1 or more antigens on the surface of glomerular podocytes, triggering an autoimmune response. IMN is a frequent glomerular illness in clinical practice, and as an autoimmune kidney disease, its pathogenesis is not yet fully understood.^[29]^

An oral drug that is frequently employed in therapeutic settings is the Huangkui capsule. Quercetin, stigmasterol, α-spinasterol, gossypetin, and β-sitosterol are 5 putative active compounds in Huangkui with OB ≥ 30% and DL ≥ 0.18 that have been found by prior network pharmacology investigations and are thought to be crucial for treating IMN. It has been demonstrated that some of these potential active ingredients have antioxidative stress, anti-inflammatory, hypotensive, anticoagulant, cholesterol-lowering, lipid-lowering, and even anticancer properties. These ingredients are currently being used in clinical settings to treat other diseases, primarily cardiovascular diseases and various inflammations.^[30–34]^ However, the extent of their use in treating renal illness is only limited, and it is still unknown whether these drugs can be modified to treat kidney disease.

According to the PPI study based on the STRING database, Huangkui capsules target numerous drug-disease common targets when treating IMN. Following screening, AKT1, MAPK3, PPARG, MMP9, ESR1, and KDR were shown to be the primary core targets. AKT1, one of the primary targets suggested for Huangkui capsule therapy for IMN, contributes to the emergence and development of IMN via the PI3K/AKT signaling pathway. AKT1 is a crucial component in this pathway and encodes serine/threonine-protein kinases. It is a member of the AKT family. It is activated by extracellular signals and takes role in boosting biological activities like metabolism, cell proliferation, cell survival, growth, and angiogenesis through a mechanism that depends on PI3K. Triptolide cures MN through modifying the P13K/AKT1/mTOR signaling pathway, according to Zhang P. et al^[35]^ network pharmacology and experimental validation, Heymann nephritis in rats was used by Yin J et al^[36]^ to show that Tetrandrine cures MN via altering the P13K/AKT signaling pathway. Furthermore, Chen J et al^[37]^ showed in vitro that Salvianolic acid B reduces MN by triggering renal autophagy via the microRNA-145-5p/P13K/AKT pathway. These findings support the findings of the KEGG pathway enrichment study and show the significance of AKT1 in the occurrence, development, and management of IMN. A crucial signaling transduction enzyme called MAPK is activated by a variety of external stimuli. It then transmits these signals to the nucleus where they promote the expression of response genes in cells.^[38]^ According to studies, betulinic acid can reduce the acute kidney damage brought on by the lysis of striated muscle in rats by preventing the phosphorylation of P38 MAPK, which involves oxidative stress, autophagy, and other processes,^[39]^ which offers a fresh method of treating renal disorders that targets MAPK. PPARG, also referred to as PPARγ, is one of the nuclear receptor superfamily’s ligand-activated transcription factors and is expressed in many kidney areas. PPARG, which has anti-fibrosis, anti-inflammatory, and antiapoptotic properties, is crucial for preserving the kidney’s normal homeostasis and function.^[40]^ MMP9 secretion by renal visceral epithelial cells was discovered to be greatly elevated in the passive Heymann nephritis (membranous nephropathy) rat model, which was connected to the development of considerable proteinuria following glomerular filtration barrier disruption.^[41]^ Additionally, it was discovered that MMP9 was considerably elevated in IMN patients” urine as compared to urine samples from healthy controls.^[42]^ ESR1 is an estrogen receptor, a transcription factor that is activated by ligands and mediates the actions of estrogen. The renal endothelin-1 system and mitochondrial homeostasis are both controlled by estrogen receptors. Through its receptor in the proximal tubule, estrogen controls phosphate balance and contributes to kidney repair and regeneration.^[43–45]^ Vascular endothelial growth factor receptor-2 is the protein that KDR encodes, and its stimulation can result in or exacerbate kidney damage.^[46]^ A VEGFR2 kinase inhibitor was used by Lavoz C et al^[47]^ to cure mouse diabetic nephropathy. The restoration of podocyte marker gene expression and the downregulation of renal injury biomarkers and pro-inflammatory mediators are examples of downstream pathways that greatly improved interstitial nephritis, tubular atrophy, and renal function. Furthermore, inhibiting VEGFR2 with pharmacological kinase inhibitors or gene silencing reversed alterations in epithelial-mesenchymal transition-related genes and decreased the activation of fibrotic factors mediated by Gremlin, delaying the onset of renal fibrosis.^[48]^

According to the findings of the GO enrichment analysis, the active components in Huangkui capsules have the potential to influence a variety of biological processes crucial to the growth of IMN, including signal transduction, protein phosphorylation, positive regulation of RNA polymerase II promoter transcription and cell proliferation, negative regulation of apoptosis, and the transmembrane receptor protein tyrosine kinase signaling pathway. An immune imbalance of the helper T (Th) cell subsets, including dominant subsets like Th2, Th17, follicular helper T cells, and subdominant subsets like regulatory T cells, which promote the occurrence and development of autoimmune reactions, is what distinguishes IMN as an autoimmune disease. One method for treating this illness is to control T cell growth.^[49]^ Nephrin is a crucial protein that makes up the glomerular filtration barrier, and it’s phosphorylation level is linked to the development of IMN proteinuria.^[50,51]^ One of the characteristics of MN is podocyte apoptosis, and some studies have suggested that preventing or even lowering podocyte apoptosis is advantageous for the treatment of MN.^[52,53]^ These existing research frameworks attest to the applicability of the GO enrichment analysis findings in this study to IMN. The key active compounds in Solanum nigrum’s treatment of IMN involve multiple signal pathways, including the PI3K-Akt signaling pathway, Endocrine resistance, Phospholipase D signaling pathway, Rap1 signaling pathway, Ras signaling pathway, and others, according to the results of KEGG pathway enrichment analysis (Fig. 7). The proliferation, differentiation, metabolism, and death of cells as well as podocyte damage, mesangial cell hypertrophy, and epithelial-mesenchymal transition of renal proximal tubular cells have all been linked to the PI3K/Akt signaling pathway.^[36]^ By decreasing the abnormally active PI3K/AKT/mTOR pathway, Terry Ting Yu Chiou et al^[53]^ have shown that rapamycin can rescue podocytes induced by PLA2R activation from the effects of apoptosis. Blocking the PI3K/Akt/mTOR signaling pathway improves autophagic activity and podocyte adhesion damage.^[54]^ In experimental membranous nephropathy rats, curcumin, an extract of the Chinese herb Curcuma, can increase renal autophagy and decrease oxidative stress by inhibiting the expression of the PI3K/Akt/mTOR signaling pathway.^[55]^ Therefore, 1 method for treating IMN may involve controlling the expression of the PI3K-Akt signaling pathway. The primary active ingredients of Huangkui capsule have regulatory effects on IMN-related molecules such AKT1, MAPK3, PPARG, MMP9, ESR1, and KDR, according to the results of molecular docking.

There are certain restrictions to this study, despite the fact that it potentially identifies the targets and signaling pathways of Huangkui capsule in the therapy of IMN. The chemical components of Huangkui that have been found are used as the research subject in network pharmacology. The advantage of this approach is that the structure and chemical constituents are both transparent, but TCM is not just a straightforward collection of chemical constituents. The concentration, content, and interactions of the various ingredients in Huangkui capsule all have an impact on their effectiveness. Additionally, the databases utilized to compile data on targets, components, etc. have limits and are unable to reveal the full scope of Huangkui capsule pharmacological effects in the management of IMN. The most crucial point is that network pharmacology research has the same limitations – any scientific hypothesis cannot be divorced from experimental verification. Only by combining network-based computational prediction and experimental confirmation, and carrying out biological experiments on the prediction results of network pharmacology of Huangkui capsules in the treatment of IMN, can the accuracy and reliability of the prediction results be verified, providing strong evidence support for the development of new Chinese medicine for treating “modern diseases”.

## 5. Conclusion

This study examined putative active ingredients and hypothesized potential molecular pathways for Huangkui capsule treatment of IMN. Six key targets for treating IMN and 5 probable active ingredients in Huangkui capsule were found in this investigation. RNA polymerase II promoter transcription and cell proliferation are positively regulated, apoptosis is negatively regulated, and tyrosine kinase signaling pathways are involved in the process by which Huangkui capsule treats IMN. Six major signaling pathways, including the PI3K-Akt signaling pathway, metabolic pathways, cancer pathways, protein glycosylation pathways in cancer, chemical carcinogenesis-receptor activation pathway, and chemical carcinogenesis-reactive oxygen species pathway, were also found to be potentially involved. Of these, the PI3K-Akt signaling pathway is the most extensively researched. The positive docking of active ingredients in Huangkui capsule with core targets was confirmed by molecular docking data. According to the findings of our study, the therapeutic impact of Huangkui capsule on IMN is attained through a variety of elements, a variety of paths, and a variety of targets. The findings of this study serve as a theoretical underpinning for the therapeutic treatment of IMN with Huangkui capsules as well as a foundation for further Huangkui capsule promotion.

## Author contributions

**Conceptualization:** Yongjing Xiang.

**Data curation:** Juan Xie.

**Funding acquisition:** Zhengsheng Li.

**Methodology:** Zhengsheng Li.

**Writing – original draft:** Meng Cai.

**Writing – review & editing:** Fulong Wen.
